# Active microneedle patch equipped with spontaneous bubble generation for enhanced rheumatoid arthritis treatment

**DOI:** 10.7150/thno.103080

**Published:** 2025-02-24

**Authors:** Ting Liu, Jintao Fu, Ziyang Zheng, Minglong Chen, Wenhao Wang, Chuanbin Wu, Guilan Quan, Xin Pan

**Affiliations:** 1 School of Pharmaceutical Sciences, Sun Yat-sen University, Guangzhou 510006, China.; 2 School of Pharmaceutical Sciences, Hainan University, Haikou 570200, China.; 3 State Key Laboratory of Bioactive Molecules and Druggability Assessment, Guangdong Basic Research Center of Excellence for Natural Bioactive Molecules and Discovery of Innovative Drugs, College of Pharmacy, Jinan University, Guangzhou 511443, China.; 4 Key Laboratory of Precision and Intelligent Chemistry, Department of Polymer Science and Engineering, School of Chemistry and Materials Science, University of Science and Technology of China, Hefei, 230001, China.

**Keywords:** transdermal drug delivery, active microneedles, bubble generation, rapid separation, rheumatoid arthritis

## Abstract

**Rationale:** The utilization of dissolving microneedles (MNs) facilitates the painless delivery of pharmaceuticals *via* the transdermal route. However, conventional MNs rely on passive diffusion through the gradual dissolving of the matrix, which can impede the therapeutic efficacy of the delivered drugs.

**Methods:** In this study, we present the development of a novel degradable active MNs platform. This platform employs sodium bicarbonate and citric acid loaded in a dissolving MNs patch as a built-in motor for deeper and faster intradermal payload delivery. The sodium bicarbonate microparticles and citric acid undergo a chemical reaction when in contact with tissue fluid, resulting in the rapid formation of explosive carbon dioxide bubbles. This provides the necessary force to break through dermal barriers and enhance payload delivery.

**Results:** The results demonstrated that the active MNs possessed excellent mechanical properties, rapid detachment characteristics, and superior drug release kinetics. Furthermore, the drug permeation behavior of active MNs exhibited enhanced permeation and distribution in skin-mimicking gel and porcine skin when compared to conventional passive MNs. *In vivo* experiments employing a rat model of rheumatoid arthritis showed that active MNs achieved superior therapeutic efficacy compared to passive MNs.

**Conclusions:** This universal and effective autonomous dynamic microneedle delivery technology is straightforward to prepare and ultilize, and has the potential to improve the therapeutic efficacy of drugs, offering significant prospects for a diverse range of therapeutic applications.

## Introduction

Rheumatoid arthritis (RA), a complex autoimmune disorder, has emerged as a major cause of disability, affecting an estimated 24.5 million individuals worldwide, with 1.2 million new cases 1. This chronic autoimmune disease is typified by joint swelling, tenderness, and symmetrical stiffness, which are caused by synovitis 2. In severe cases, the disease can cause irreversible damage to cartilage and bone tissue, leading to joint deformity and disability 3. Moreover, RA exerts a systemic impact on vital organs, including the heart, lungs, and blood vessels. Consequently, precise and personalized management is a pivotal aspect of patient care, facilitating the improvement of clinical symptoms, the postponement of disease progression, and the mitigation of disability. Common treatment drugs include disease-modifying antirheumatic drugs (DMARDs), anti-inflammatory agents, and analgesic medications 4, which are typically administered orally or by injection in clinical practice 5. However, oral drugs are often poorly tolerated due to gastrointestinal side effects 6 and injectable therapies carry risks of adverse reactions such as nephrotoxicity and hepatotoxicity 7. Given these challenges, researchers have sought to identify alternative routes of drug delivery for the RA treatment 8. Transdermal drug delivery system (TDDS) represents an appealing option, as it circumvents the first-pass effect inherent to oral drugs and simultaneously mitigates the risks and inconveniences of injection administration 9-12.

In TDDS, drug permeability is a key performance indicator directly influencing therapeutic efficacy. Techniques such as penetration enhancers, microjets, and iontophoresis have been developed to overcome the stratum corneum barrier 13, but these methods are often costly and impractical 14. Dissolving microneedles (MNs) offer a simpler alternative by creating micropores in the skin, improving drug delivery 15-17 while avoiding contact with deep dermal nerves and blood vessels, thereby enhancing safety and compliance 18-20. However, traditional MNs rely on passive diffusion, which limits drug penetration depth and distribution. Active interventions, such as electroporation 21, temperature modulation 22,23, and light activation 24,25, can improve delivery efficiency but require bulky equipment, reducing their practicality. Another challenge with traditional MNs is their prolonged wear time, which depends on material dissolution rate. This extended duration can decrease patient compliance and cause skin irritation 26. Existing efforts to address these challenges have focused on designs that enable rapid separation of MNs from the patch base, including multi-layer MNs and bubble-containing MNs 27-31. For instance, we previously developed bilayer MNs with a polymer-sugar complex that disintegrated within 30 seconds, facilitating rapid detachment of drug-concentrated tips from the base 32. Similarly, Li *et al.* reported MNs with an air bubble between the needle and patch backing to enable quick detachment upon insertion 33. While these approaches successfully reduce wear time, they lack active drug delivery capabilities and involve complex fabrication processes.

An ideal active MN system for RA treatment should integrate two critical features: (1) active drug delivery with cost-effective manufacturing to enhance penetration and efficiency, and (2) rapid separation to minimize application time. Furthermore, rapid drug release in active MNs is essential for arthritis treatment, as it provides prompt symptom relief, suppresses inflammation, quickly achieves therapeutic drug concentrations, and meets clinical demands for immediate availability during flare-ups. Therefore, future efforts should focus on developing MN platforms that combine active delivery and rapid separation in a simple, efficient, and cost-effective manner to optimize therapeutic outcomes.

**Figure 1:**
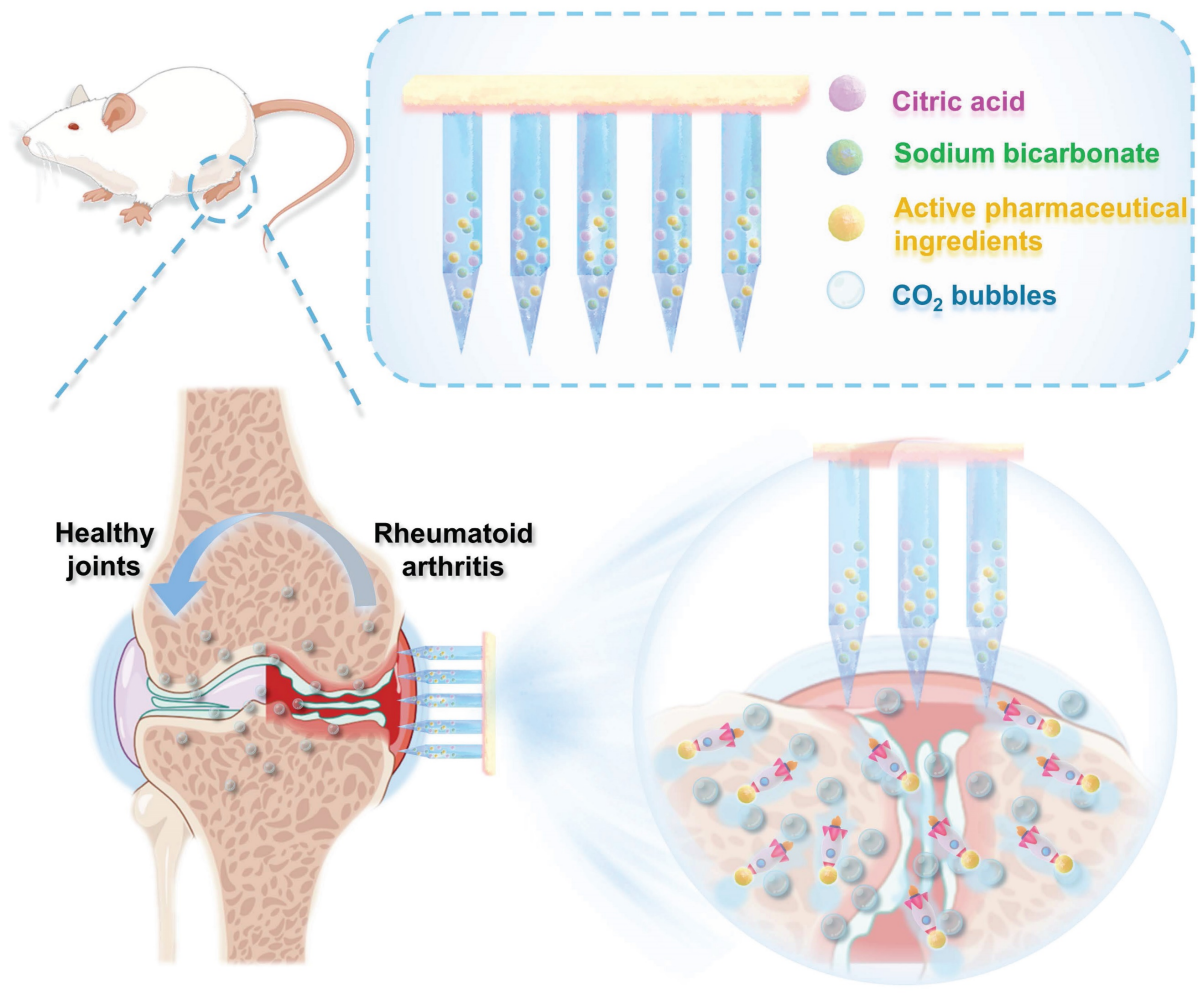
Schematic illustration of the effervescent active MNs with spontaneous bubbles generation for enhanced drug delivery in RA treatment.

Here, we developed a biodegradable active MN delivery platform that spontaneously generates bubbles, thereby greatly enhancing drug permeation (Figure 1). Concurrently, the drug-loaded needle is rapidly separated from the base upon contact with the skin. The MN patch consists of a biodegradable polymer loaded with the therapeutic payload along with an effervescent formulation. Upon insertion, the MNs contact interstitial biofluid within the skin. The sodium bicarbonate and citric acid in the needle then undergo an instantaneous chemical reaction, resulting in the generation of carbon dioxide bubbles. The spontaneous generation of bubbles induces a powerful “micromotor” effect, resulting in dynamic transport and enhanced permeation of the embedded payload. The formation of microbubbles also results in the weakening of the needle's attachment to the base, thereby enabling rapid separation upon insertion into the skin. The active MN patch was prepared and characterized in terms of its mechanical behavior, rapid detachment properties, and drug release kinetics. The drug permeation behavior was observed* in vitro* through the detection of the fluorescence intensity change in skin-mimicking gel and porcine skin, indicating an enhanced permeation and distribution compared to that observed with common passive MNs. The advantages of the active MN patch in drug delivery and therapy were validated using an adjuvant-induced arthritis (AIA) rat model. Methotrexate (MTX), a first-line treatment for RA, was chosen as the model drug, and the active MNs-MTX group demonstrated enhanced therapeutic efficacy compared to passive MNs. Overall, the effervescent active MNs delivery system presented here provides spontaneous bubble generation, rapid separation, and faster release of the payload through the skin, offering a form of transdermal administration with high compliance and superior therapeutic efficacy.

## Experimental Section

### Materials and animals

Sodium bicarbonate was obtained from Macklin Biochemical Technology Co., Ltd. (Shanxi, China). MTX and citric acid were purchased from Aladdin Industrial Ltd. (Shanghai, China). Polyvinyl pyrrolidone (PVP) K30 and K90 were kindly donated by BASF (Ludwigshafen, Germany). Polydimethylsiloxane (PDMS) was purchased from Dow Corning Co., Ltd. (Michigan, USA). Complete Freud's Agent (CFA) was purchased from Chondrex (Washington DC, USA). EDTA decalcifying solution was obtained from Servicebio (Hubei, China). Antibodies used for immunofluorescence staining were purchased from Servicebio (Hubei, China).

The healthy male Sprague-Dawley (SD) rats weighing 220-250 g were purchased from the Laboratory Animal Center of Sun Yat-sen University (Guangzhou, China). All animal experiments were carried out following the guidelines for the care and use of laboratory animals under the approval of the Laboratory Animal Center of Sun Yat-sen University (Guangzhou, China). The rats were adapted to the environment for 3 days before the start of the experiment, and standard food and water were provided throughout the experiments.

### Preparation of MNs

The active MNs were fabricated by the micro-molding strategy through a centrifugation method 34. To prevent premature bubble formation in aqueous environments, anhydrous ethanol was selected as the solvent. This choice imposed stricter requirements on the dissolution of polymer materials and the preparation process. Briefly, a mixture of sodium bicarbonate (50 mg·mL^-1^), citric acid (70 mg·mL^-1^), PVP K30 (150 mg·mL^-1^), and PVP K90 (150 mg·mL^-1^) in ethanol was filled into the surface of the PDMS female mold and filled into micro molds under centrifugation at 4000 rpm for 5 min at 4 °C. After removal of the excess polymer suspension, the molds were dried in a sealed container for 24 h. This procedure was repeated three times. Subsequently, the PVP K90 ethanol solution (300 mg·mL^-1^) was poured onto the mold, followed by centrifugation under the same conditions. Finally, the mold was dried again for 48 h, and the MNs patch was carefully peeled from the female mold. The preparation of passive MNs was similar to that of active MNs without adding sodium bicarbonate and citric acid.

### Characterization of MNs

Bubble generation and dissolving behavior: A MNs patch was immersed in 1 mL of phosphate buffer saline (PBS) and observed using optical microscope to study the bubble generation and dissolving behavior of MNs. The samples were imaged at predetermined time intervals (0, 5, 10, 20, 30, and 60 s).

COMSOL multiphysics simulation: A transient laminar flow model was used in COMSOL to simulate the flow process of payload in water. First, a 2D geometry was constructed and unstructured triangular meshes were created. The size of the meshes was controlled by physical field and there were 3222 meshes in total. The diffusion coefficient of gas in water was set as 0.0016 mm²·s^-1^. Next, the flow rate of bubbles was 2×10^-4^ kg (m^2^·s)^-1^. Finally, the transient flow process with a total duration of 10 s and time step of 0.1 s was calculated.

Mechanical strength and diffusion behavior: The mechanical strength of MNs was tested using the excised rat skin *via* hematoxylin-eosin (H&E) staining. Additionally, MNs were manually pushed into gelatin as a simulated skin and observed using confocal laser scanning microscope (CLSM, FV3000, Olympus, Japan) to assess the diffusion efficiency of MNs. The same operation was performed with pig skin in lieu of gelatin.

### *In vitro* drug release test

To observe the *in vitro* release profiles of MTX from MNs, a MN array (1 cm × 1 cm) was immersed in 1 mL of PBS and incubated at 37 °C. Samples were collected at predetermined time intervals (0, 1, 2, 3, 4, 5, 10, 20, 30, 60, and 90 min), and the medium was replaced with the same volume of fresh release medium. The amount of released MTX was determined by high-pressure liquid chromatography (HPLC, LC-20AT, Shimadzu, Japan) and the experiment was conducted in triplicate.

### *In vivo* retention study

To better evaluate drug retention behavior in the skin, live whole animal imaging was carried out by an *in vivo* imaging system (IVIS) (Night OWL II LB983, BERTHOLD, German). The fluorescence images of the MN-treated sites at specific time points were recorded.

### *In vivo* therapeutic effect

All animal experiments were performed with the approval of the Animal Ethics Committee of Sun Yat-sen University (SYSU-IACUC-2022000139). AIA rats were induced through subcutaneous injection of Complete Freud's Agent (CFA, 100 μL) containing 10 mg·mL^-1^ of heat-killed mycobacteria (Chondrex, Washington DC, USA) into the rats' right footpad. In the normal control group, normal saline (100 μL) was injected according to the same protocol. AIA rats were randomly divided into six groups, and the treatment details of each group are given as follows:

Group 1: Normal rats (administered normal saline)

Group 2: AIA rats (immunized with CFA)

Group 3: AIA rats treated with free MTX solution by oral administration (Oral-MTX)

Group 4: AIA rats treated with free MTX solution by subcutaneous injection (SC-MTX)

Group 5: AIA rats treated with MTX loaded passive microneedle at the rats' right footpad (Passive MNs-MTX)

Group 6: AIA rats treated with MTX loaded active microneedle at the rats' right footpad (Active MNs-MTX)

Treatment groups were administered (equivalent to 750 μg·kg^-1^) every 2 days for a total of 7 times, respectively. The clinical arthritis scoring criteria were measured according to the standard scale as reported 35,36: 0, no erythema or swelling; 1, mild erythema and swelling confined to the ankle joint; 2, mild erythema and swelling extending from the ankle to the tarsus; 3, moderate erythema and swelling; Swelling extending to the metatarsal joints; 4, severe erythema and swelling encompassing the ankle and the entire paw. During treatment, rats were examined every 2 days since the first administration to measure the clinical arthritis score according to the above criteria. The right hind paw thickness and the dimensions of ankle joints of every rat were measured with a vernier caliper before each administration.

### Radiological analysis of hind paws

On the 28th day, all groups' collected ankle joints were subjected to micro-computed tomography (micro-CT, Bruker Skyscan 1176). The micro-CT images of the right hind paw of each rat group were taken to examine the degree of swelling and bone erosion.

### Histological and immunohistochemical analysis of hind paws

Right ankle joints and hind paws were collected from the mice on the 28th day and stored in 4% phosphate buffer paraformaldehyde for 48 h and completely decalcified by incubation with EDTA decalcification solution. Decalcified tissues were placed in paraffin, then thin cross sections were made using a cryostat and stained with hematoxylin and eosin (H&E), safranin O-fast green, and TNF-α. Finally, the images were obtained using a light microscope.

### Safety evaluation

As described in “*In vivo* therapeutic effect”, the blood samples were collected *via* retro-orbital bleeding before the sacrifice of the rats. The serum was obtained by centrifugation for 15 min at 3000 rpm, and then blood cell counts, serum levels of alanine aminotransferase (ALT), aspartate aminotransferase (AST), blood urea nitrogen (UREA), and creatinine (CREA) were determined.

Furthermore, the rats with different treatments were sacrificed and the major organs (heart, liver, spleen, lung, and kidney) were collected and fixed with 4% paraformaldehyde. After being embedded in paraffin, the organs were sliced for H&E staining.

### Statistical analysis

The statistical analyses were performed using student's t-test or one-way analysis of variance (ANOVA) test by GraphPad Prism 9.0.0. Differences were considered statistically significant at *P < 0.05.

## Results and Discussion

### Preparation and characterization of MNs

**Figure 2:**
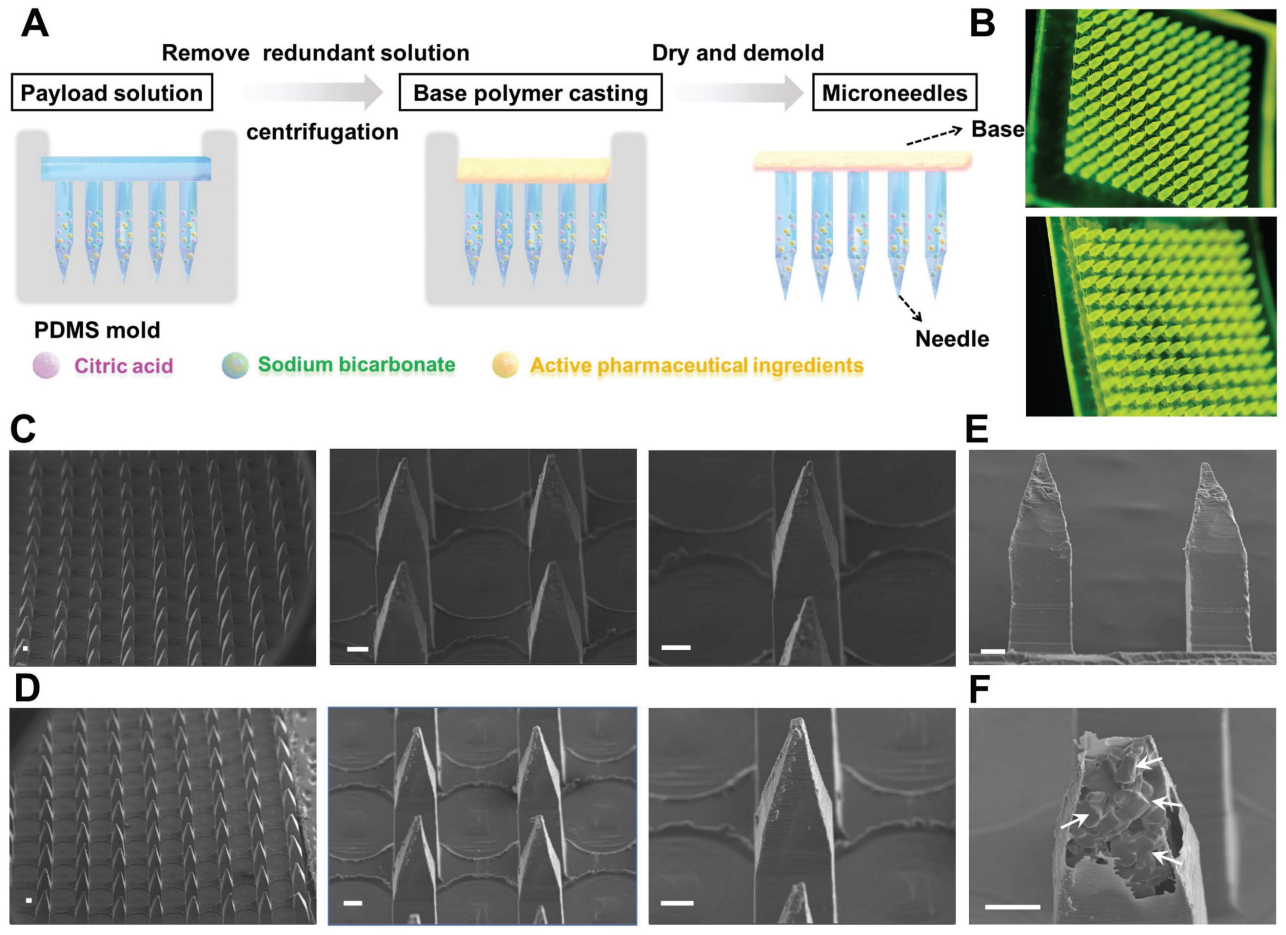
** Preparation and characterizations of active MNs.** (A) Schematic illustration of the preparation process of active MNs. (B) The optical handheld microscopy images of active MNs (upper) and passive MNs (lower) loaded with coumarin 6. (C, D) SEM images of active MNs (upper) and passive MNs (lower). (E) SEM image of the sideview of active MNs. (F) Magnified SEM image of inside active MNs (Scale bar = 100 μm).

To achieve rapid separation of the MN patch, an effervescent formulation (sodium bicarbonate and citric acid) was employed in combination with biodegradable polymer and therapeutic agent to prepare an active MN delivery platform. This design allows for rapid separation of the active MN patch from the base while enhancing drug permeation through bubble generation. The active MNs were fabricated by a micromolding strategy through a centrifugation method as previously described (Figure 2A). The results observed by handheld optical microscopy showed that both active MNs and passive MNs (without effervescent formulation) exhibited a 12 × 12 microneedle array (Figure 2B), demonstrating that the structure of active MNs remains unaltered by the incorporation of effervescent microparticles. Scanning electron microscopy (SEM) images of the MNs revealed that the active MNs possessed an intact hard structure and maintained a sharp tip comparable to that of the passive MNs (Figure 2C-D). Each microneedle consisted of a quadrilateral prism base (300 μm × 300 μm) and a quadrilateral cone, with a height of 1200 μm. The overall dimensions of the microneedle were carefully designed to optimize the balance between structural integrity and effective penetration into the skin. As illustrated in Figure 2E-F, the magnified SEM image of a single active MN tip demonstrated that the embedded effervescent microparticles were clearly visible within the microneedle protected by the exterior polymer, further confirming the successful construction of the active MNs.

### The distribution of ingredients in MNs

**Figure 3:**
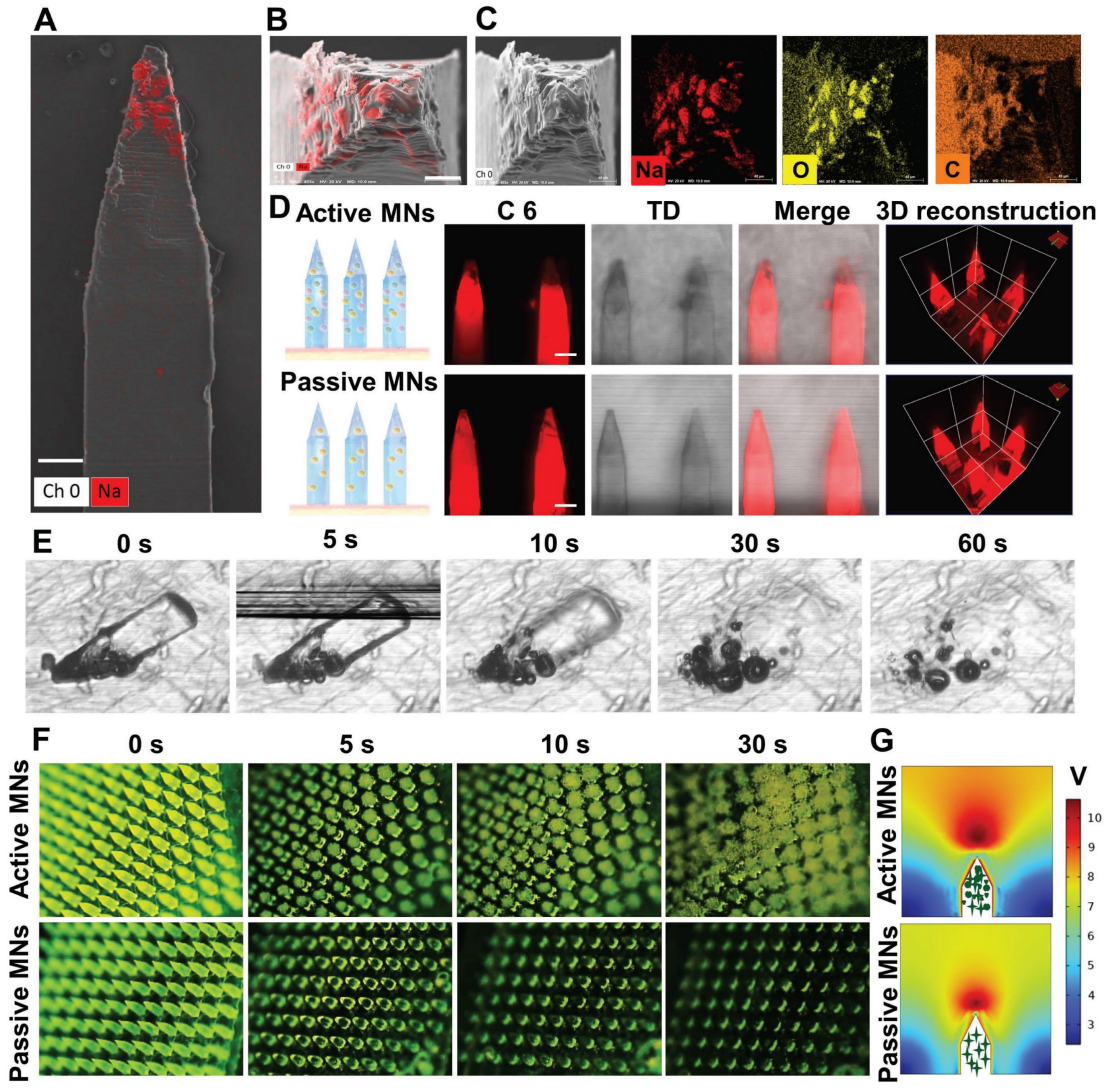
** The ingredient distribution and bubble generation behavior of MNs.** EDX analysis of the side view (A) (Scale bar = 90 μm) and the top view (B) (Scale bar = 40 μm) of active MNs. (C) SEM images of single active MN tip and EDX analysis for sodium (Na), oxygen(O), and carbon (C). (D) CLSM and 3D reconstruction images of C6-loaded MNs (Red signal, scale bar = 200 μm). (E) Time-lapse images of single active MNs, showing bubble production and polymer dissolving in PBS. (F) Time-frame images of MNs, showing bubble production and drug diffusion in PBS. (G) COMSOL Multiphysics simulation of the flow generated by active MNs and passive MNs.

The distribution of effervescent microparticles and therapeutic payload in a single microneedle was characterized by energy dispersive X-ray spectroscopy (EDX) mapping and CLSM. The results of t Scanning electron microscope showed that sodium and oxygen were uniformly dispersed at the tip of the active MN (Figure 3A-C). The 3and Three-dimensional (3D) reconstruction images of the MNs demonstrated that the loaded drug was uniformly distributed within the MNs, revealing that the addition of effervescent microparticles did not alter the drug distribution within the active MNs (Figure 3D).

### The dissolving and bubble generation behavior of active MNs

The capacity to generate bubbles was of paramount importance to achieve rapid dissolving of active MNs. Therefore, the dissolving and bubble generation behaviors of MNs were investigated by microscopic observation and COMSOL multiphysics simulation. As illustrated in Figure 3E, the active MNs underwent rapid dissolving in contact with fluids, with complete dissolving occurring within 30 s. Additionally, continuous bubble production was observed within 60 s. It is clearly depicted that the bubbles were rapidly generated around the embedded active microparticles, which led to an explosive-like behavior (Figure 3F). The accelerated dissolving and enhanced mixing induced by bubble production were further corroborated by a COMSOL multiphysics simulation. The flow induced by active MNs was more vigorous than that induced by passive MNs (Figure 3G), which was consistent with the results in Figure 3E and F. Once the active effervescent ingredients were in contact with fluids, bubbles were substantially generated and promoted local mixing and flow motion.

### The affect and dissolving behavior of MTX-loaded active MNs

**Figure 4:**
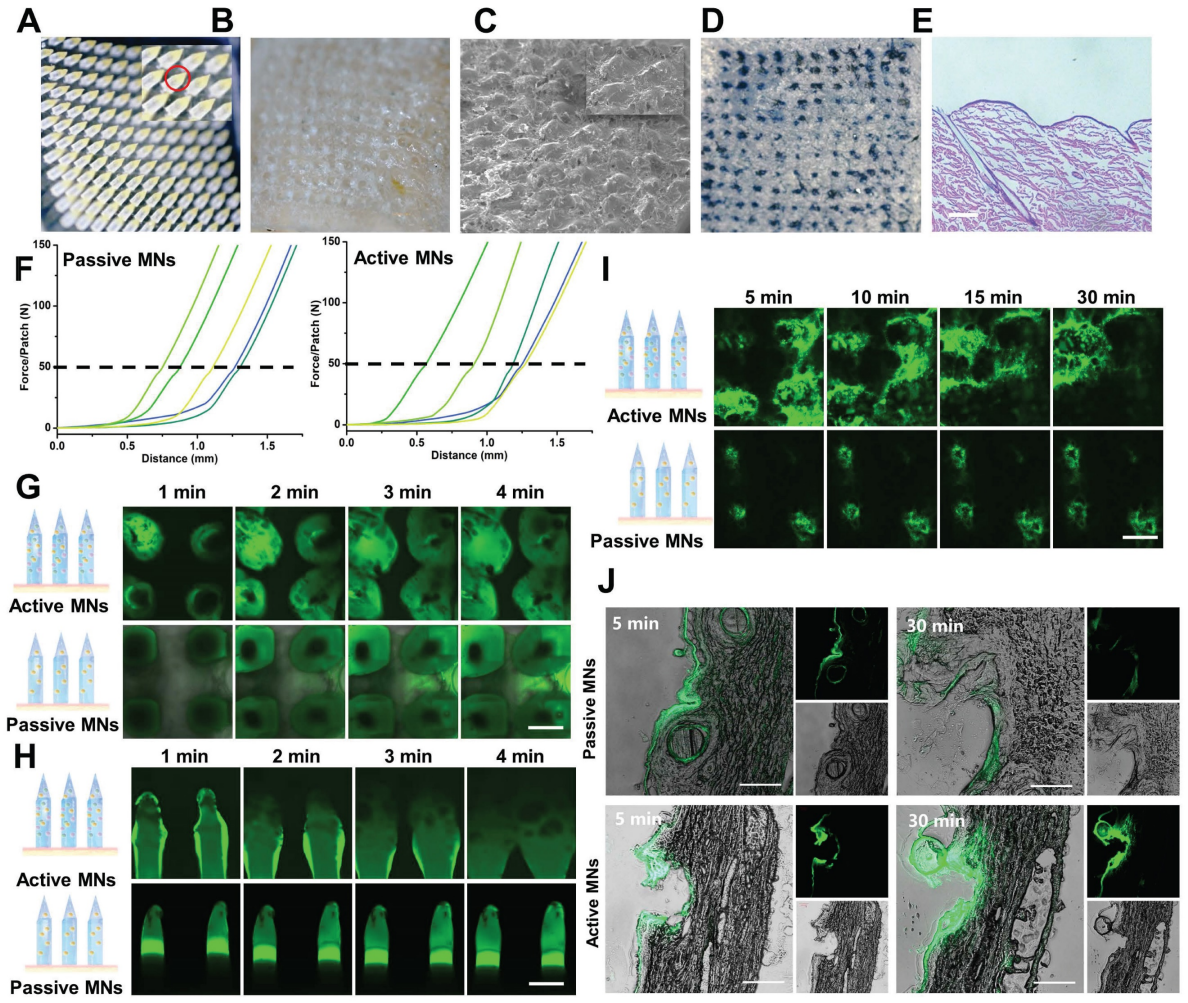
** Mechanical strength and diffusion behavior of active MNs.** (A, B) Images of active MNs before (A) and after (B) application to isolated rat skin. (C) SEM image of active MNs after application to isolated rat skin (Scale bar = 700 μm). (D) The photograph of the rat skin after active MNs insertion. (E) The H&E staining image of the rat skin sections after active MNs insertion (Scale bar = 50 μm). (F) The force curve of active MNs and passive MNs detected by the texture analyzer (*n* = 5). Time-lapse CLSM top view (G) and side view (H) images of active MNs and passive MNs inserted in gelatin-simulated skin, obtained at different time points (0-4 min). (I) Time-lapse CLSM top view images of porcine skin after application of active MNs and passive MNs at different time points (5-30 min). (Scale bar = 400 μm). (J) Fluorescence images of porcine skin cryosections after application of active MNs and passive MNs for 5 and 30 min. (Scale bar = 100 μm).

MTX, a first-line drug for RA treatment, was selected as a model drug to assess the efficacy of active MNs. MTX is typically administered orally or by injection 37. However, long-term systemic exposure to MTX is associated with dose-dependent toxicity 38. The use of MNs can offer a safer and more convenient alternative to the current administration routes of MTX 39,40. As shown in Figure 4A, the yellow part represents the loaded MTX, which is predominantly distributed at the tip of the active MNs. It is noteworthy that MNs necessitate excellent mechanical properties to enable utilization without breaking. To corroborate the ability of MNs to be inserted into the skin, the MTX-loaded active MNs were applied to isolated rat abdominal skin. Following a three-minute application, the base of the MN patch could be completely peeled away from the skin (Figure 4B and C). Subsequently, trypan blue staining was employed to visualize the microchannels within the MNs-punctured skin, and the results showed that regular microholes could be clearly observed corresponding to the microneedle array (Figure 4D). The histological section of the inserted skin displayed clear micropores with a depth of approximately 400 μm, indicating that the MTX-loaded active MNs possessed sufficient mechanical strength to successfully pierce the *stratum corneum* (Figure 4E). As shown in Figure 4F, the active MNs achieved a fracture force of approximately 50 N during compression, similar to that of passive MNs. This result indicated that the incorporation of effervescent microparticles did not affect the mechanical strength of the active MNs. In addition, the dissolving behavior of MNs was monitored by examining their morphological changes during application to the skin. The active MNs were observed to dissolve at a significantly accelerated rate, with the dissolving process occurring within 90 seconds (Figure S1). This indicates that the incorporation of active ingredients could facilitate the separation of MNs, consequently reducing the requisite time for microneedle application to the skin 41. This outcome was primarily attributed to the reaction of citric acid and sodium bicarbonate in active MNs, which resulted in the formation of carbon dioxide bubbles and subsequent weakening of the adhesion of MNs to the base.

### Horizontal and vertical diffusion of active MNs

The diffusion of MNs in skin was evaluated by measuring the permeation of the coumarin 6 molecule using CLSM. Initially, the horizontal and vertical diffusion of both active MNs and passive MNs were examined in gelatin-simulated skin *via* top and side view fluorescence (Figure 4G-H). A comparison was conducted between the active MNs and passive MNs, and it was observed that the active MNs contained opaque effervescent microparticles. The top view images, captured at varying time intervals, revealed that the accelerated horizontal diffusion of the active MNs could be discerned within 4 min, as evidenced by an augmented fluorescence radius of diffusion (Figure 4G). Moreover, the diffusion depth of both active MNs and passive MNs was also corroborated by side-view images. The results showed that the fluorescence surrounding the active MNs was more pronounced and extensive (Figure 4H). Furthermore, following the application of the active MNs to an isolated porcine skin, the fluorescent area exhibited a significant diffusion within 30 min compared to that of passive MNs (Figure 4I), which further validated the results presented in Figure 4G. Fluorescence imaging of porcine skin cryosections and 3D distribution analysis showed that the fluorescent areas of active MNs exhibited more pronounced diffusion depth at both 5 and 30 min compared to passive MNs, confirming that active MNs effectively enhanced the vertical diffusion of the payload within the skin (Figure 4J and Figure S2). These findings substantiate the assertion that the active MNs markedly enhance the diffusion and permeability of the payload in skin, a phenomenon attributed to the generation of CO_2_ bubbles, in contrast to the diminished delivery observed with passive MNs.

### *In vitro* drug release and *in vivo* permeation profiles of MNs

**Figure 5:**
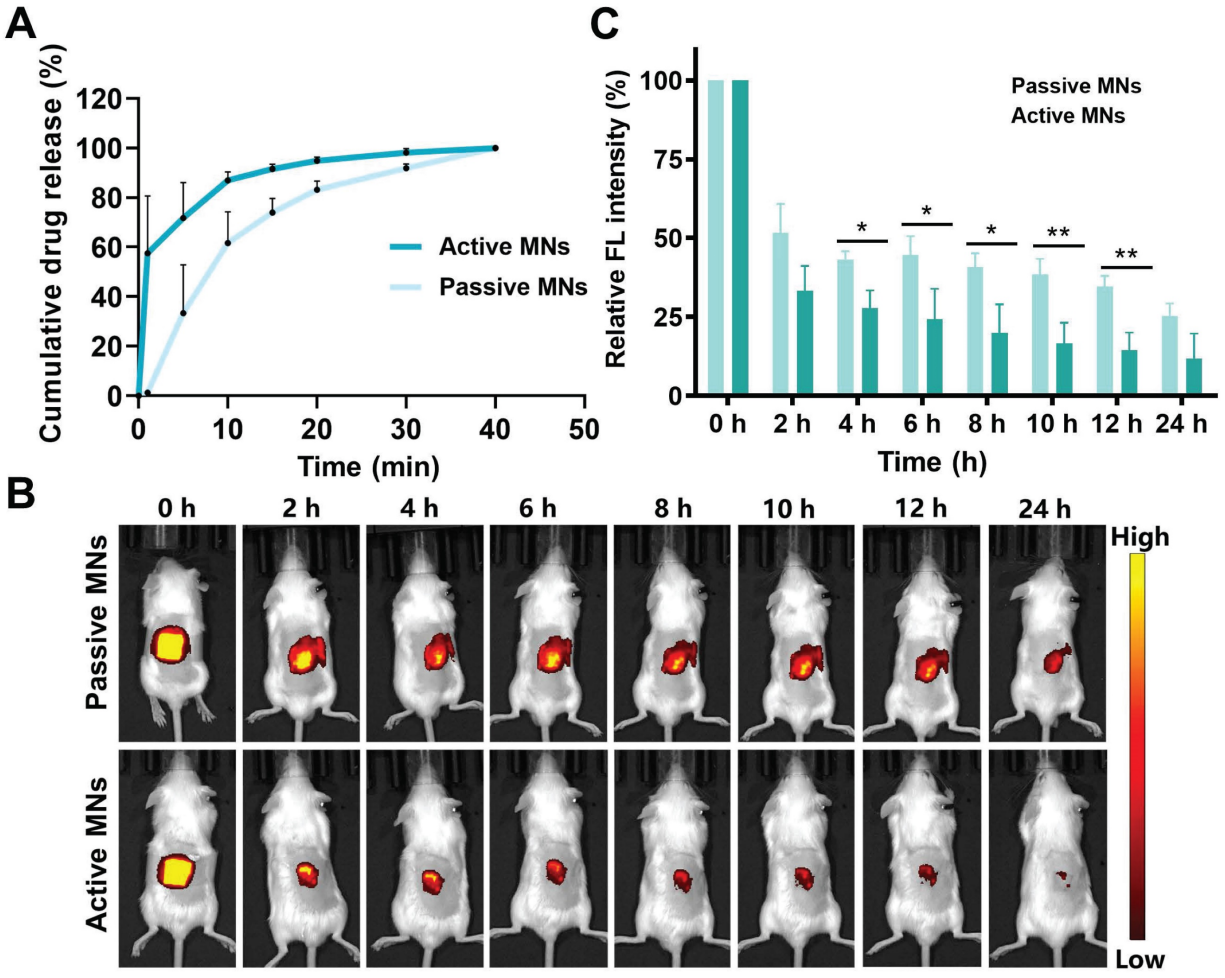
***In vitro* drug release and *in vivo* permeation profiles of MNs.** (A) Cumulative *in vitro* release profiles of MTX from MNs. (B) *In vivo* fluorescence images of mice treated with active MNs and passive MNs and (C) its semiquantitative result.

The MTX was released from the MNs over a period of 40 min following the placement of the MN in PBS at 37 °C (Figure 5A). The quantitative *in vitro* release profiles of MTX clearly demonstrated that active MNs exhibited a superior performance compared to passive MNs. At the one-minute mark, the passive diffusion approach exhibited a release percentage of 1.2 ± 0.8%, while the active MN delivery method resulted in a significantly higher release of 57.5 ± 18.9%. The rapid release of MTX from active MNs was ascribed to the expeditious generation of CO_2_ and H_2_O by the reaction of sodium bicarbonate and citric acid, which powered the immediate delivery of embedded therapeutic ingredients and consequently accelerated MN hydration. Furthermore, the dissolving of effervescent agents resulted in the formation of a porous structure, which increased the contact area between the MNs and the medium, thereby also accelerating the release process.

To investigate the transcutaneous localization and release pattern, *in vivo* fluorescence images of the treated site were obtained by an IVIS to ascertain the drug retention behavior of both active and passive MNs (Figure 5B-C). The results demonstrated that the fluorescence intensity of the active MNs was equivalent to that of the passive MNs at the initial time point. However, the subsequent fluorescence signal exhibited a gradual decline at all indicated time points in comparison to passive MNs. This phenomenon may be attributed to the rapid dissolution rate and drug permeation profile, indicating that the released drug was effectively eliminated from the insertion site. Subsequently, the recovery state of the skin barrier function was evaluated by recording the skin after active MN administration at different time intervals (Figure S3). The results indicated that the punctured skin probably recovered its integrity at 30 min, and the complete return of the skin to its normal state was observed at 2 h. Altogether, these findings confirmed an improved drug release profile, faster permeation, and the elimination of loaded cargo within active MNs in comparison to passive MNs. Thus, we postulated that the expeditious release of MTX at cutaneous arthrosis lesions would serve to augment the therapeutic efficacy of the treatment.

### *In vivo* therapeutic effects in AIA rats

**Figure 6:**
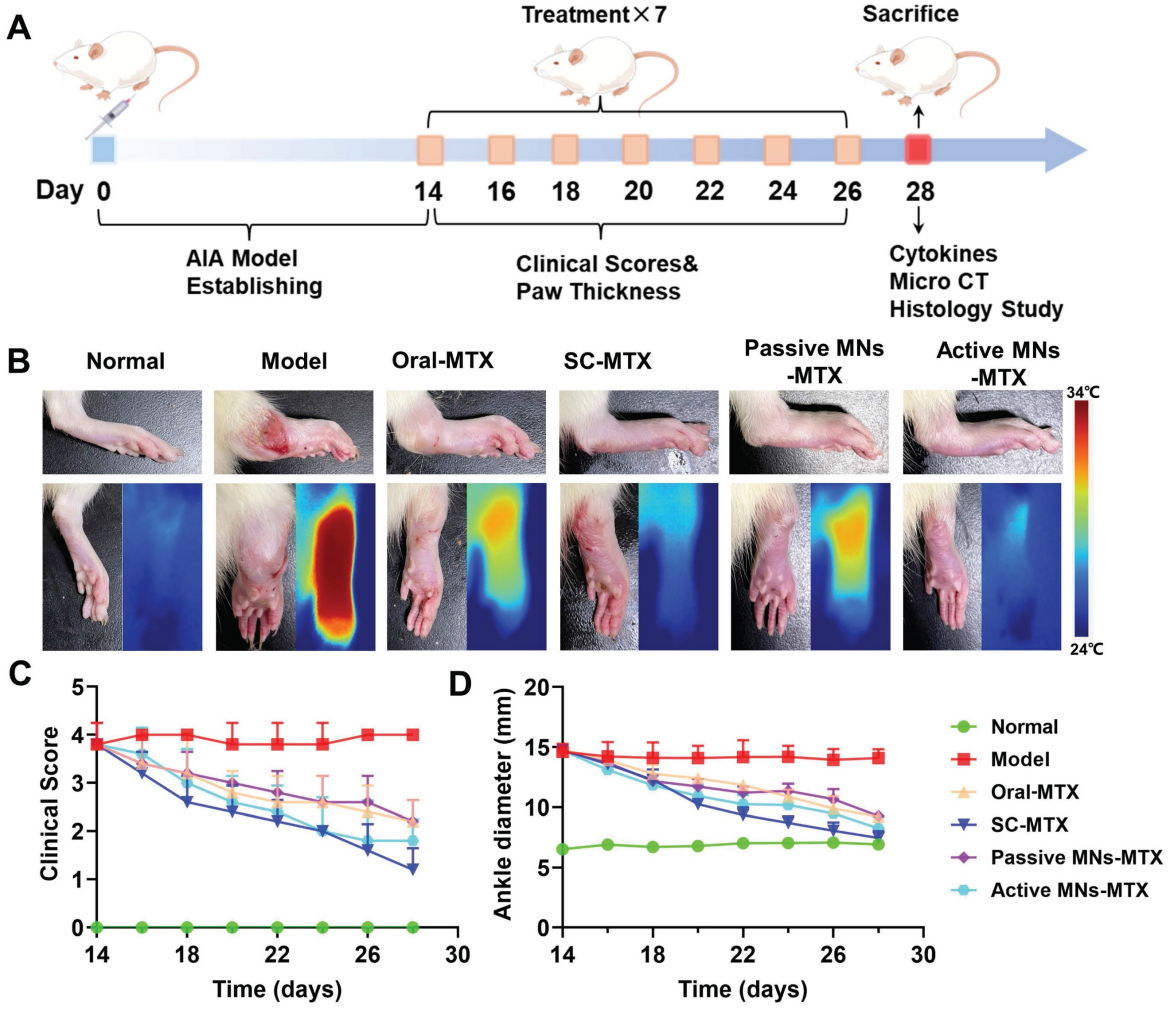
** Inhibitory effect of MTX-loaded active MNs on arthritic progression in AIA rats.** (A) Schematic illustration of the treatment protocol for AIA rats. (B) Representative hind paw images of the normal, model, oral-MTX, SC-MTX, passive MNs-MTX, and active MNs-MTX groups. (C) Clinical scores of AIA rats in different groups. (D) Ankle diameter change of AIA rats in different groups. Data are shown as the mean ± SD (*n* = 5).

In order to assess the inhibitory effect of MTX-loaded active MNs on the progression of arthritis, an AIA model was established in rats. As the treatment regimen outlined in Figure 6A, the MTX-loaded active and passive MNs were applied topically to AIA rats, while free MTX was administered orally and subcutaneously to serve as a control. During the treatment period, the AIA rats were evaluated for clinical arthritis scoring at two-day intervals, commencing with the initial administration and concluding after seven assessments. The hind paw thickness and ankle joint dimensions were measured in each group of rats prior to each administration. The rats were executed after completion of treatment, and the therapeutic efficacy was also evaluated through the analysis of cytokine levels, micro-CT imaging, and histologic examination. The severity of arthritis was indicated by paw thickness and clinical scores. A notable increase in the thickness of the hind paw and ankle joint was observed in the model rats in comparison to healthy rats (Figure 6B). The MTX-loaded passive MNs could partially decrease the swelling paw thickness by 36.9%. By contrast, AIA rats treated with MTX-loaded active MNs exhibited a significant reduction in paw swelling thickness of 43.9%, indicating that active MNs could offer a more effective approach to controlling joint inflammation than passive MNs. The therapeutic efficacy of MTX-loaded active MNs was found to be most closely aligned with that of SC MTX, exhibiting a 49.4% reduction in swelling paw thickness (Figure 6D). This outcome not only validates the effectiveness of active MNs as a transdermal drug route but also underscores its potential as an optimal route for the treatment of articular inflammation. A similar pattern was observed in the clinical scoring data (Figure 6C). The anti-arthritic efficacy of passive MNs and oral administration was moderate, as evidenced by a reduction in clinical score. In contrast, MTX-loaded active MNs exhibited lower clinical scores, second only to SC MTX. These results revealed the efficacy of MTX-loaded active MNs in reducing paw swelling in AIA rats.

### Bone erosion in the ankle tissue

**Figure 7:**
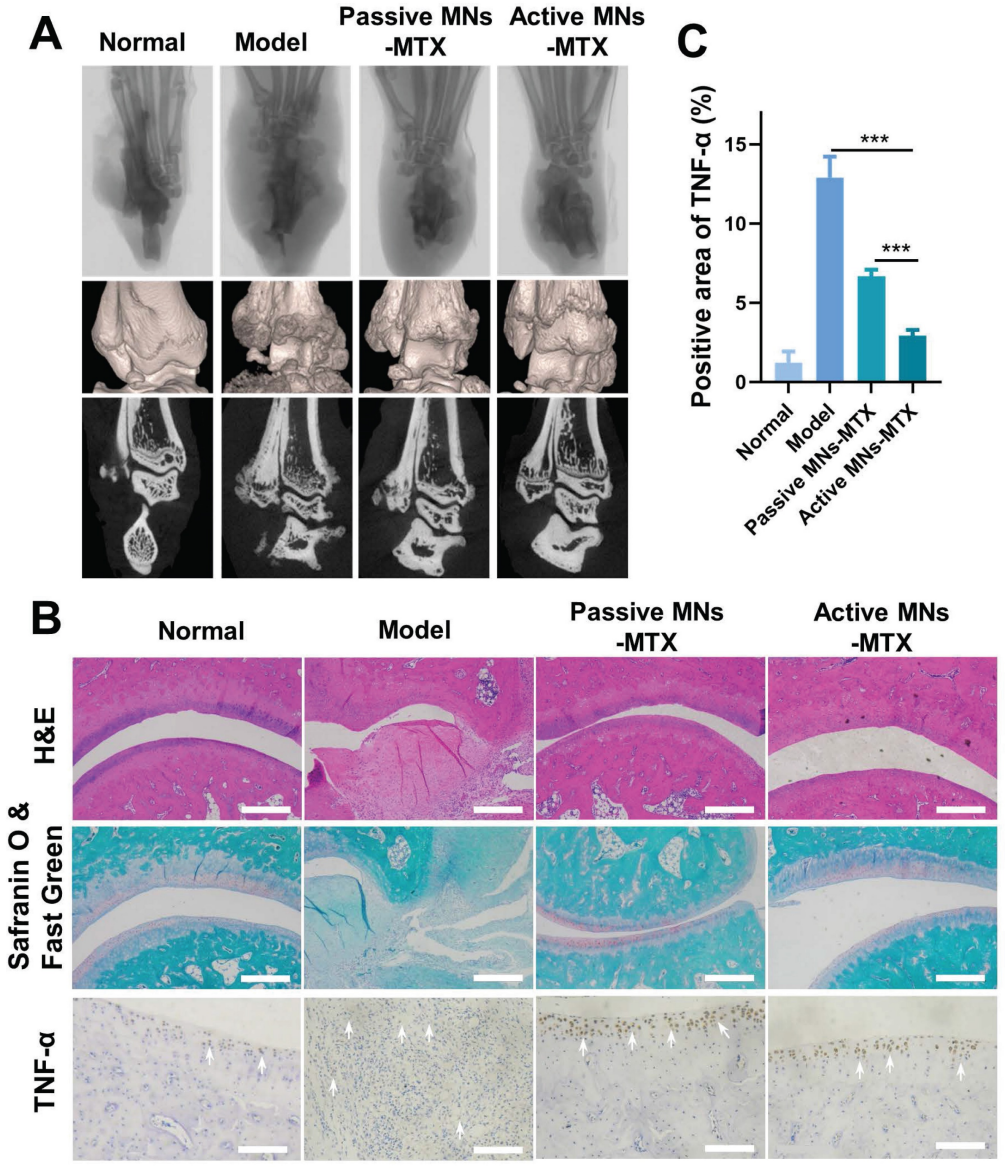
** The effect of active MNs-MTX on relieving bone erosion, arthritic inflammation, and cartilage damage.** (A) Representative images of Micro-CT imaging assessment. (B) Histological and immunohistochemical analysis of hind paws after various treatments. (Scale bar = 100 μm). (C) Semiquantitative analysis of inflammatory cytokine expression in joint tissues. Data are shown as the mean ± SD (*n* = 3).

Since bone erosion is a major symptom of severe RA, micro-CT was used to investigate the impact of active MNs on bone erosion in inflamed ankle joints (Figure 7A and Figure S4A). Compared with normal rats, the articular surfaces of the ankles of model rats revealed rough bone surfaces and obvious bone erosion. Oral MTX, SC MTX, and passive MNs-MTX group showed moderate therapeutic efficacy against bone destruction in AIA rats.

Notably, minimal bone erosion and cartilage damage was observed in the active MNs treatment group. This indicates that the generation of bubbles and the rapid separation, along with the induction of a faster payload release, could serve as an effective method for protecting bones from destruction and erosion. To further substantiate the therapeutic efficacy of active MNs, a histological analysis of arthritic inflammation and cartilage destruction was conducted on the ankle joints of AIA rats from different groups (Figure 7B and Figure S4B). In contrast to healthy rats, the model rats demonstrated distinct indications of inflammatory cell infiltration on the articular surface, as evidenced by the results of H&E-staining. The overall condition of inflammatory cell infiltration was improved to a certain extent in each treatment group. Specifically, the active MNs treatment displayed superior efficacy in joint and synovial recovery, exhibiting minimal pathological features. In addition, the ankle joint was stained using Safranin O&FastGreen, which stains glycosaminoglycans in cartilage 42. The results demonstrated clear staining and well-defined borders in the normal group, while the model group exhibited uneven staining and faded coloration, indicative of substantial cartilage damage. However, following treatment with the active MNs, staining results similar to those observed in the normal group were noted, thereby proving that active MNs are capable of effectively maintaining cartilage integrity. In order to further prove the inflammatory status after different treatments, the expression of pro-inflammatory cytokines (TNF-α) was analyzed *via* immunohistochemistry. It was observed that the group treated with active MNs exhibited a relatively low expression of TNF-α compared to the other groups. The semiquantitative analysis of inflammation indicators demonstrated that the normal group exhibited negligible cytokine expression in the joint tissues of rats, while the model group showed a substantial increase in TNF-α levels, indicating severe inflammation (Figure 7C). Treatment with active MNs-MTX significantly reduced cytokine expression compared to the model group and passive MNs-MTX groups, achieving values close to the normal group. To summarize, the results demonstrated that this intervention with active MNs significantly contributes to the mitigation of damage to the articular cartilage and bone, as well as the reduction of pro-inflammatory cytokine levels. These findings substantiate the efficacy of the active MNs in controlling joint inflammation and offer a promising avenue for RA therapy.

### Safety evaluation

**Figure 8:**
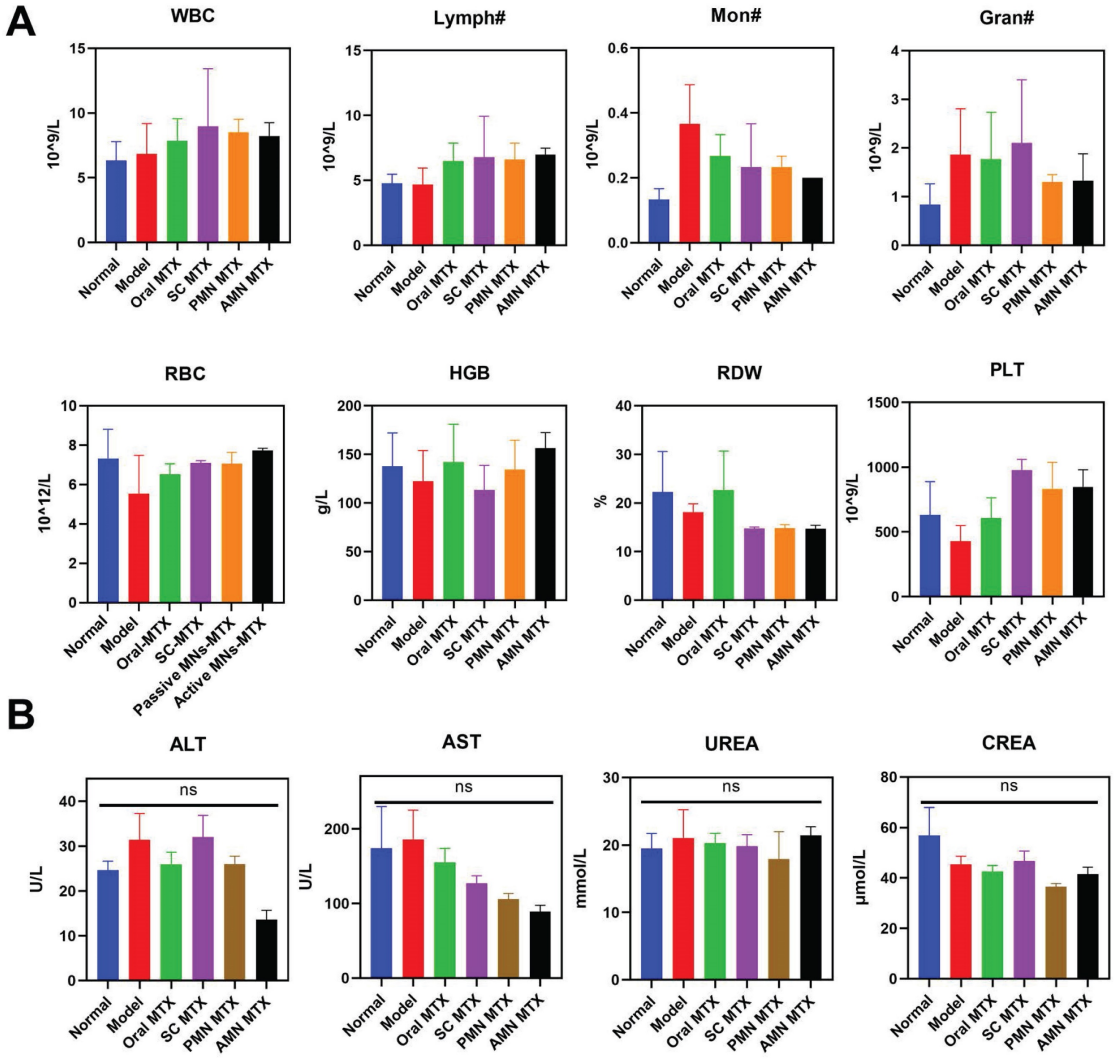
** Safety Evaluation.** (A) Hematologic parameters of all experimental groups on day 16 (*n* = 3). (B) Serum biochemistry results of treated AIA rats (*n* = 3).

To ascertain the safety of the treated AIA rats, an investigation was conducted following the different routes of administration. This involved routine blood analysis, serum biochemical analysis, and H&E pathological observation of major organs. As shown in Figure 8A, the hematological parameters in the active MNs-treated AIA rats remained within normal physiological ranges: white blood cell count (WBC): 2.9-15.3 10^9/L, lymphocyte count (Lymph#): 2.6-13.5 10^9/L, monocyte count (Mon#): 0.0-0.5 10^9/L, granulocyte count (Gran#): 0.4-3.2 10^9/L, red blood cell count (RBC): 5.6-7.89 10^12/L, hemoglobin (HGB): 120-150 g/L, red blood cell volume distribution width (RDW): 11-15.5 %, platelet count (PLT): 100-1610 10^9/L. Furthermore, the levels of ALT, AST, UREA, and CREA showed no statistically significant differences between the active MNs-treated AIA rats and the normal rats (Figure 8B). The histological analysis of the major organs (heart, liver, spleen, lung, and kidney) for the active MNs-treated AIA rats also displayed no obvious damage in comparison to the normal rats (Figure S5). Based on these results, it is reasonable to conclude that the active MNs represent a promising alternative strategy for the efficacious treatment of RA.

## Discussion and Conclusion

Dissolving MNs offer a painless, transdermal route for drug delivery, but traditional MNs rely on passive diffusion and gradual matrix dissolution, which can limit the therapeutic efficacy of delivered drugs. In this study, we successfully developed an innovative active MNs delivery platform, integrating therapeutic drug loading with an effervescent formulation to enhance the treatment of RA. The unique mechanism of spontaneous carbon dioxide bubble generation upon insertion provides dual functionality: (1) enabling dynamic and efficient intradermal drug delivery with deeper penetration and faster release, and (2) facilitating rapid detachment of the MNs from the patch base, thereby reducing application time and improving patient compliance. The platform demonstrated excellent mechanical properties, rapid separation characteristics, and superior drug release kinetics. The *in vitro* permeation studies revealed that active MNs significantly improved drug delivery efficiency, showing enhanced drug permeation and distribution in both skin-mimicking gels and porcine skin. Furthermore, *in vivo* studies using an AIA rat model validated the superior therapeutic efficacy of active MNs, demonstrating enhanced alleviation of RA symptoms compared to conventional passive MNs. Importantly, the safety evaluation confirmed the biocompatibility of the system, with no notable adverse effects on major organs or hematological parameters, highlighting its suitability for long-term use.

Beyond the scope of RA treatment, this active MN platform holds significant potential for broader biomedical applications. The effervescent bubble-driven mechanism offers a versatile and cost-effective strategy that can be adapted for the delivery of various therapeutic agents, including biologics, vaccines, and gene therapies. Its dynamic delivery approach is particularly promising for diseases requiring localized, rapid, and efficient drug delivery, such as chronic inflammatory conditions (e.g., psoriasis) or localized tumors. Additionally, the platform's straightforward fabrication process and autonomous operation make it highly feasible for large-scale production and point-of-care applications.

Future investigations should focus on optimizing the effervescent formulation for different drug types, assessing its long-term therapeutic outcomes, and exploring its application in other preclinical disease models. Overall, the proposed active MN system offers a transformative approach to transdermal drug delivery, combining enhanced therapeutic efficacy with improved patient convenience, and holds considerable promise for revolutionizing the treatment of a wide range of diseases.

## Supplementary Material

Supplementary figures.

Supplementary info: video of active MNs for bubble production.

Supplementary info: video of passive MNs for bubble production.

## References

[1] Liu Y, Xie W, Tang Z, Tan Z, He Y, Luo J (2024). A reconfigurable integrated smart device for real-time monitoring and synergistic treatment of rheumatoid arthritis. Sci Adv.

[2] Hua P, Liang R, Yang S, Tu Y, Chen M (2024). Microneedle-assisted dual delivery of PUMA gene and celastrol for synergistic therapy of rheumatoid arthritis through restoring synovial homeostasis. Bioact Mater.

[3] Liu T, Fu J, Chen M, Wu Q, Quan G, Wu C (2024). *In situ* polymeric nanomicelle-generating dissolving microneedle patch for enhanced transdermal methotrexate delivery in rheumatoid arthritis treatment. Eur Polym J.

[4] Xie J, Zhu X, Wang M, Liu C, Ling G, Zhang P (2024). Dissolving microneedle-mediated transdermal delivery of flurbiprofen axetil-loaded pH-responsive liposomes for arthritis treatment. Chem Eng J.

[5] Nooreen R, Nene S, Jain H, Prasannanjaneyulu V, Chitlangya P, Otavi S (2022). Polymer nanotherapeutics: a versatile platform for effective rheumatoid arthritis therapy. J Control Release.

[6] Zhao Y, Chen X, He P, Wang X, Xu Y, Hu R (2024). Transdermal microneedles alleviated rheumatoid arthritis by inducing immune tolerance via skin-resident antigen presenting cells. Small.

[7] Zheng L, Chen Y, Gu X, Li Y, Zhao H, Shao W (2024). Co-delivery of drugs by adhesive transdermal patches equipped with dissolving microneedles for the treatment of rheumatoid arthritis. J Control Release.

[8] Du G, He P, Zhao J, He C, Jiang M, Zhang Z (2021). Polymeric microneedle-mediated transdermal delivery of melittin for rheumatoid arthritis treatment. J Control Release.

[9] Lin Y, Chen Y, Deng R, Qin H, Li N, Qin Y (2023). Delivery of neutrophil membrane encapsulated non-steroidal anti-inflammatory drugs by degradable biopolymer microneedle patch for rheumatoid arthritis therapy. Nano Today.

[10] Hu H, Ruan H, Ruan S, Pei L, Jing Q, Wu T (2022). Acid-responsive PEGylated branching PLGA nanoparticles integrated into dissolving microneedles enhance local treatment of arthritis. Chem Eng J.

[11] Chen K, Zhao Y, Zhao W, Mao X, Li D, Wang Y (2024). Lubricating microneedles system with multistage sustained drug delivery for the treatment of osteoarthritis. Small.

[12] Xia T, Zhu Y, Li K, Hao K, Chai Y, Jiang H (2024). Microneedles loaded with cerium-manganese oxide nanoparticles for targeting macrophages in the treatment of rheumatoid arthritis. J Nanobiotechnology.

[13] Lee H, Song C, Baik S, Kim D, Hyeon T, Kim DH (2018). Device-assisted transdermal drug delivery. Adv Drug Deliv Rev.

[14] Sabbagh F, Kim BS (2022). Recent advances in polymeric transdermal drug delivery systems. J Control Release.

[15] Pan X, Quan G, Wu C, Chen M, Yang D, Sun Y (2021). *In situ* self-assembly nanomicelle microneedles for enhanced photoimmunotherapy via autophagy regulation strategy. ACS Nano.

[16] Yang D, Chen M, Sun Y, Shi C, Wang W, Zhao W (2023). Microneedle-assisted vaccination combined with autophagy regulation for antitumor immunotherapy. J Control Release.

[17] Zhou Y, Niu B, Zhao Y, Fu J, Wen T, Liao K (2021). Multifunctional nanoreactors-integrated microneedles for cascade reaction-enhanced cancer therapy. J Control Release.

[18] Wu B, Fu J, Zhou Y, Luo S, Zhao Y, Quan G (2020). Tailored core-shell dual metal-organic frameworks as a versatile nanomotor for effective synergistic antitumor therapy. Acta Pharm Sin B.

[19] Lin S, Quan G, Hou A, Yang P, Peng T, Gu Y (2019). Strategy for hypertrophic scar therapy: Improved delivery of triamcinolone acetonide using mechanically robust tip-concentrated dissolving microneedle array. J Control Release.

[20] Qin W, Quan G, Sun Y, Chen M, Yang P, Feng D (2020). Dissolving microneedles with spatiotemporally controlled pulsatile release nanosystem for synergistic chemo-photothermal therapy of Melanoma. Theranostics.

[21] Choi SO, Kim YC, Park JH, Hutcheson J, Gill HS, Yoon YK (2010). An electrically active microneedle array for electroporation. Biomed Microdevices.

[22] Lee H, Choi TK, Lee YB, Cho HR, Ghaffari R, Wang L (2016). A graphene-based electrochemical device with thermoresponsive microneedles for diabetes monitoring and therapy. Nat Nanotechnol.

[23] Lee H, Song C, Hong YS, Kim M, Cho HR, Kang T (2017). Wearable/disposable sweat-based glucose monitoring device with multistage transdermal drug delivery module. Sci Adv.

[24] Chen MC, Lin ZW, Ling MH (2016). Near-infrared light-activatable microneedle system for treating superficial tumors by combination of chemotherapy and photothermal therapy. ACS Nano.

[25] Dong L, Li Y, Li Z, Xu N, Liu P, Du H (2018). Au nanocage-strengthened dissolving microneedles for chemo-photothermal combined therapy of superficial skin tumors. ACS Appl Mater Interfaces.

[26] Li W, Tang J, Terry RN, Li S, Brunie A, Callahan RL (2019). Long-acting reversible contraception by effervescent microneedle patch. Sci Adv.

[27] Zhu DD, Wang QL, Liu XB, Guo XD (2016). Rapidly separating microneedles for transdermal drug delivery. Acta Biomater.

[28] Yin Y, Su W, Zhang J, Huang W, Li X, Ma H (2021). Separable microneedle patch to protect and deliver DNA nanovaccines against COVID-19. ACS Nano.

[29] Wang QL, Zhu DD, Liu XB, Chen BZ, Guo XD (2016). Microneedles with controlled bubble sizes and drug distributions for efficient transdermal drug delivery. Sci Rep.

[30] Wang H, Fu Y, Mao J, Jiang H, Du S, Liu P (2022). Strong and Tough Supramolecular Microneedle Patches with Ultrafast Dissolution and Rapid-Onset Capabilities. Adv Mater.

[31] Yu K, Yu X, Cao S, Wang Y, Zhai Y, Yang F (2021). Layered dissolving microneedles as a need-based delivery system to simultaneously alleviate skin and joint lesions in psoriatic arthritis. Acta Pharm Sin B.

[32] Hou A, Quan G, Yang B, Lu C, Chen M, Yang D (2019). Rational design of rapidly separating dissolving microneedles for precise drug delivery by balancing the mechanical performance and disintegration rate. Adv Healthc Mater.

[33] Li W, Terry RN, Tang J, Feng MR, Schwendeman SP, Prausnitz MR (2019). Rapidly separable microneedle patch for the sustained release of a contraceptive. Nat Biomed Eng.

[34] Yang B, Dong Y, Shen Y, Hou A, Quan G, Pan X (2021). Bilayer dissolving microneedle array containing 5-fluorouracil and triamcinolone with biphasic release profile for hypertrophic scar therapy. Bioact Mater.

[35] Ma Y, Lu Z, Jia B, Shi Y, Dong J, Jiang S (2022). DNA origami as a nanomedicine for targeted rheumatoid arthritis therapy through reactive oxygen species and nitric oxide scavenging. ACS Nano.

[36] Qindeel M, Khan D, Ahmed N, Khan S, Asim Ur Rehman (2020). Surfactant-free self-assembled nanomicelles-based transdermal hydrogel for safe and targeted delivery of methotrexate against rheumatoid arthritis. ACS Nano.

[37] Khan ZA, Tripathi R, Mishra B (2012). Methotrexate: a detailed review on drug delivery and clinical aspects. Expert Opin Drug Deliv.

[38] Solomon DH, Glynn RJ, Karlson EW, Lu F, Corrigan C, Colls J (2020). Adverse effects of low-dose methotrexate: a randomized trial. Ann Intern Med.

[39] Bi D, Qu F, Xiao W, Wu J, Liu P, Du H (2023). Reactive oxygen species-responsive gel-based microneedle patches for prolonged and intelligent psoriasis management. ACS Nano.

[40] Wu C, Cheng J, Li W, Yang L, Dong H, Zhang X (2021). Programmable polymeric microneedles for combined chemotherapy and antioxidative treatment of rheumatoid arthritis. ACS Appl Mater Interfaces.

[41] Zhao E, Xiao T, Tan Y, Zhou X, Li Y, Wang X (2023). Separable microneedles with photosynthesis-driven oxygen manufactory for diabetic wound healing. ACS Appl Mater Interfaces.

[42] Kim J Y, Rhim W K, Lee S Y, Park J M, Song D H, Cha S G (2024). Hybrid nanoparticle engineered with transform-ing growth factor-β1-overexpressed extracellular vesicle and cartilage-targeted anti-inflammatory liposome for osteoarthritis. ACS Nano.

