# Supplementary material for: Active microneedle patch equipped with spontaneous bubble generation for enhanced rheumatoid arthritis treatment
Source: Theranostics. 2025 Feb 24;15(8):3424–38. doi: 10.7150/thno.103080 (PMC11905133; doi:10.7150/thno.103080)
Supplement: Supplementary file 1 — Supplementary figures. [file thnov15p3424s1.pdf]

# Supporting Information

## Active microneedle patch equipped with spontaneous bubble generation for enhanced rheumatoid arthritis treatment

Ting Liu[a,†], Jintao Fu[b,†,*], Ziyang Zheng[c], Minglong Chen[d], Wenhao Wang[a], Chuanbin Wu[c], Guilan Quan[c,*], Xin Pan[a,*]

[a] *School of Pharmaceutical Sciences, Sun Yat-sen University, Guangzhou 510006, China*

[b] *School of Pharmaceutical Sciences, Hainan University, Haikou 570200, China*

[c] *College of Pharmacy, Jinan University, Guangzhou 511443, China*

[d] *Key Laboratory of Precision and Intelligent Chemistry, Department of Polymer Science and Engineering, School of Chemistry and Materials Science, University of Science and Technology of China, Hefei, 230001, China.*

[*] Corresponding authors.

E-mail addresses: fujintao@hainanu.edu.cn (J. Fu), quanguilan@jnu.edu.cn (G. Quan), panxin2@mail.sysu.edu.cn (X. Pan).

[†] Ting Liu and Jintao Fu contributed equally to this paper.

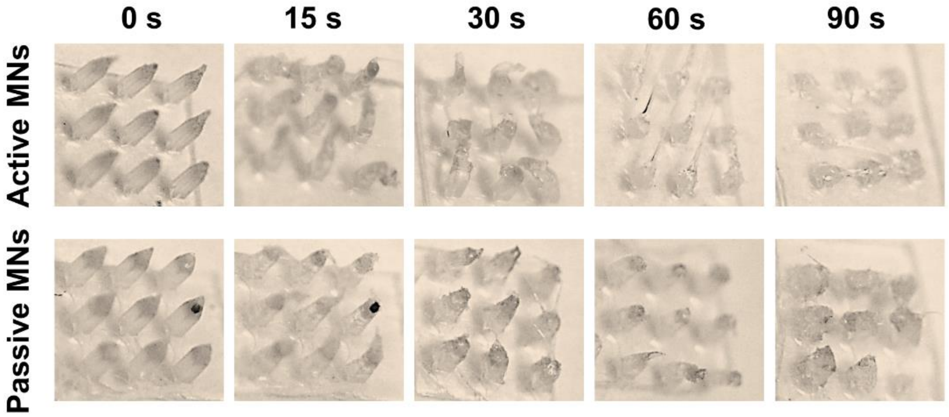


Figure S1. The photographs of active MNs and passive MNs after application to gelatin-simulated skin.

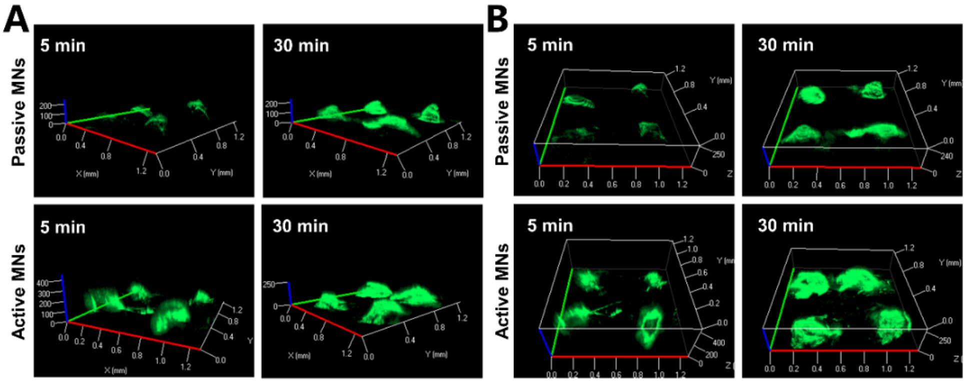


Figure S2. Three-dimensional distribution of active MNs and passive MNs inserted in pig skin for 5 and 30 min in different fields of view.

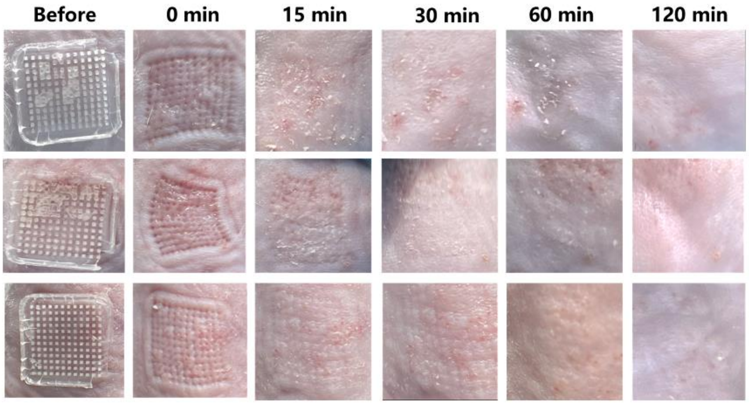


Figure S3. Representative images of mice skin after application of an active MNs in vivo at different time intervals.

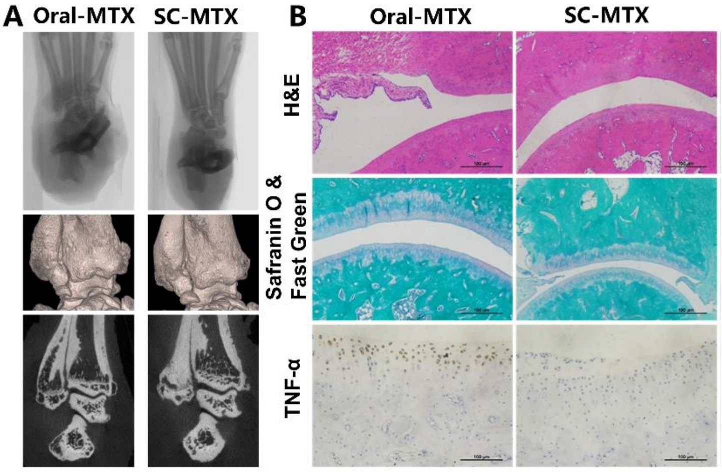

Figure S4. (A) Representative images of Micro-CT imaging assessment of oral-MTX group and SC-MTX group. (B) Histological and immunohistochemical analysis of hind paws in oral-MTX group and SC-MTX group.

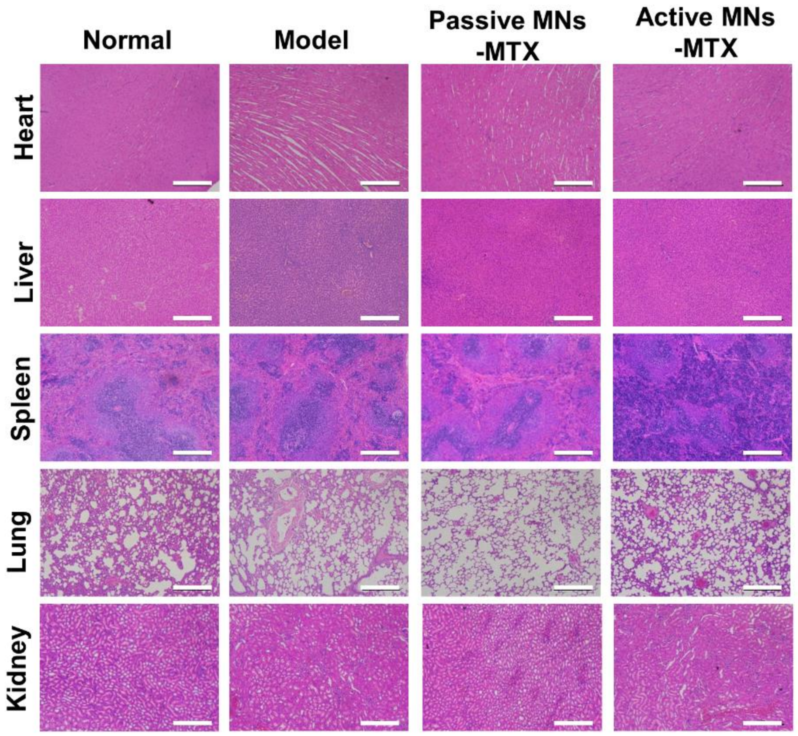


Figure S5. Representative H&E staining of major organs in different treated AIA rats (Scale bar = 100 μm).
